# Supplementary material for: Easy Synthesis and Characterization of Novel Carbon Dots Using the One-Pot Green Method for Cancer Therapy
Source: Pharmaceutics. 2022 Nov 9;14(11):2423. doi: 10.3390/pharmaceutics14112423 (PMC9696114; doi:10.3390/pharmaceutics14112423)
Supplement: Supplementary file 1 [file pharmaceutics-14-02423-s001.zip › pharmaceutics-1971143-supplementary.pdf]

Supplementary Materials

Easy synthesis and characterization of novel carbon dots using the one-pot green method for cancer therapy

Experimental Section

S1. Fluorescence spectra of the CD_{C-H} at different mass ratios

The excitation and emission spectra of the CD_{C-H} at different CMCS to mass ratios were investigated by using a Spectra Max M3 microplate reader (USA). Fluorescence emission spectra were scanned at the excitation wavelength of 357 nm for carbon dot solutions with different mass ratios and equal concentration configurations. A fixed emission wavelength of 467 nm was used to scan the fluorescence excitation spectra of carbon dot solutions with different mass ratios.

S2. Loading efficiency (LE) and loading capacity (LC) of DOX-CD_{C-H}

The fluorescence intensity at different time points was measured by Spectra Max M3 microplate reader (E_x/E_m: 495/590 nm) to estimate the loading efficiency (LE) and loading capacity (LC) of DOX. The DOX-CD_{C-H} solution was dialyzed against into the deionized water at room temperature in darkness for 2 h to remove unloaded DOX molecules. The final products in the dialysis bag were freeze-dried for further analysis. The loading efficiency and loading content of DOX were calculated as follows equation(Eq.S1 and Eq.S2):

$$LE(\%) = (W_{DOX} - W_{free})/W_{DOX\text{-}total} \times 100\% \quad (S1)$$

$$LC(\%) = (W_{DOX} - W_{free})/W_{CD_{C-H}} \times 100\% \quad (S2)$$

where $W_{CD_{C-H}}$ represents the total quantity of CD_{C-H}; $W_{DOX\text{-}total}$ is the total amount of DOX in the reaction.

S3. *In vitro* cytotoxicity assay

The *in vitro* cytotoxicity of CD_{C-H} against NIH-3T3, MCF-7 and 4T1 cells was investigated using the MTT method. The detailed operation process can be summarized as follows: firstly, cells were seeded in 96-well plates (100 μL/well) at a density of $1\sim2\times10^4$ cells/well and incubated in a 5% CO_2 at a temperature of 37 ˚C for 24 h. Subsequently, the medium was removed and samples at different concentrations were replaced and then again incubated for 24 h and 48 h. After incubation, 10 μL of MTT (5 mg/mL) solution was added to each well and incubation at 37 ˚C for an additional 4 h. After removing that culture medium, 200 μL of DMSO was added to fully dissolve the crystal of formazan, and the absorbance of each well was estimated at 490 nm using SpectraMax M3 microplate reader (USA).

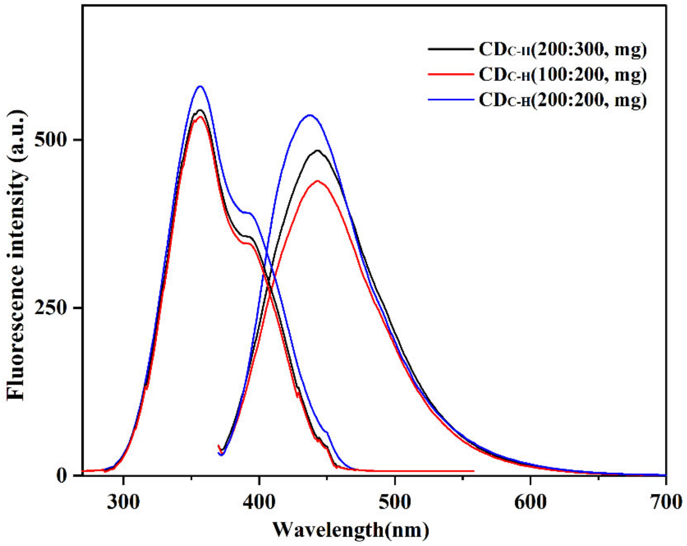

Figure S1. The excitation and emission spectra of the CD_{C-H} at different CMCS to mass ratios.

Table S1. Hydrate particle sizes (PS) and particle size distribution index (PDI), loading efficiency (LE) and loading content (LC) of DOX-CD$_{C-H}$ complexes. Values are mean ± SD (n = 3).

Sample	LC (%)	LE (%)	Hydrate Particle Sizes	PDI
DOX-CD$_{C-H}$	36.73 ± 1.98	6.11 ± 1.60	201.06 ± 4.92	0.23 ± 0.041

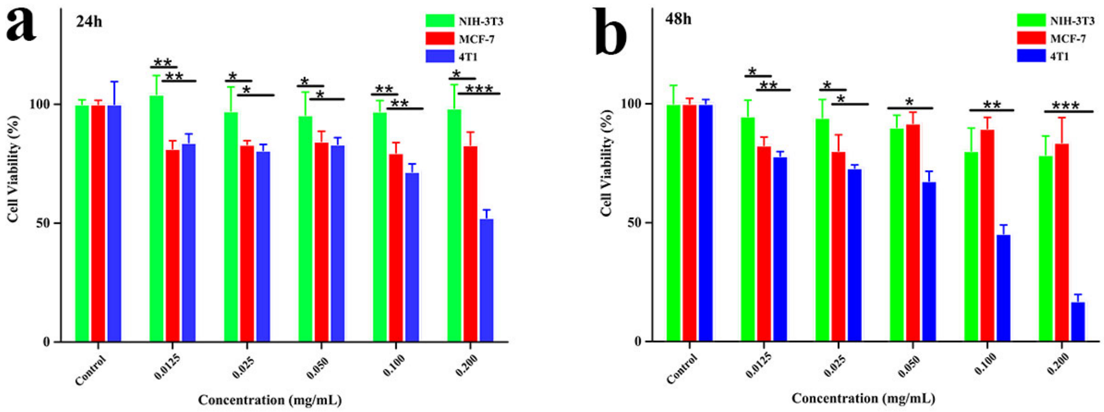

Figure S2. Cytotoxicity results of the CD$_{C-H}$ to NIH-3T3, MCF-7 and 4T1 cells at 24h (**a**) and 48h (**b**); Data represent mean ± SD, n = 3; *p < 0.05, **p < 0.01 and ***p < 0.001, *vs.*NIH-3T3 cells.
